# A Hidden Markov Model for Copy Number Variant prediction from whole genome resequencing data

**DOI:** 10.1186/1471-2105-12-S6-S4

**Published:** 2011-07-28

**Authors:** Yufeng Shen, Yiwei Gu, Itsik Pe’er

**Affiliations:** 1 Department of Computer Science, Columbia University, New York, New York 10027, USA; 2 Center for Computational Biology and Bioinformatics, Columbia University, New York, New York 10032, USA

## Abstract

**Motivation:**

Copy Number Variants (CNVs) are important genetic factors for studying human diseases. While high-throughput whole genome re-sequencing provides multiple lines of evidence for detecting CNVs, computational algorithms need to be tailored for different type or size of CNVs under different experimental designs**.**

**Results:**

To achieve optimal power and resolution of detecting CNVs at low depth of coverage, we implemented a Hidden Markov Model that integrates both depth of coverage and mate-pair relationship. The novelty of our algorithm is that we infer the likelihood of carrying a deletion jointly from multiple mate pairs in a region without the requirement of a single mate pairs being obvious outliers. By integrating all useful information in a comprehensive model, our method is able to detect medium-size deletions (200-2000bp) at low depth (<10× per sample). We applied the method to simulated data and demonstrate the power of detecting medium-size deletions is close to theoretical values.

**Availability:**

A program implemented in Java, *Zinfandel*, is available at http://www.cs.columbia.edu/~itsik/zinfandel/

## Introduction

An important utility of whole-genome resequencing (WGS) is to systematically uncover structural variations (SVs) including copy number variants (CNVs). There are two major approaches to infer SVs from resequencing data. The first one is to align the reads onto a reference genome and then infer the SVs from the reads alignment. The second one is to *de novo* assemble the reads into larger genomic fragments (contigs or scaffolds) and then infer the SVs by aligning the fragments to a reference genome. Most of the current high-throughput sequencing platforms achieve efficiency by generating massive amount of short paired-end reads in a single run [1,2], making it much easier to map the reads to a reference [3-7] than to carry out *de novo* assembly. Here we focus on the first approach.

In general there are two major sources of information useful for inferring SVs from reads alignment: depth of coverage and break points. Depth of coverage positively correlates with the copy number [8,9]. Break points, marking the boundary of SVs, can be inferred from sequence alignment gaps, which are suitable for short insertion/deletion (indel) discovery [3,10], or mate-pair distance abnormally, which is usually the basis of detecting large structural variations [11,12]. Most existing tools only utilize one type of evidence, even though these two are complementary. On the one hand, depth of coverage provides more reliable information for large CNVs or CNVs flanked by repeats, where accurate mapping of reads around breakpoints is difficult. On the other hand, breakpoints provide better power to detect small- (through sequence alignment gaps) to medium- size (through mate pair abnormally) indels or CNVs. In particular, mate pairs from relatively large inserts provide larger physical depth of coverage than sequence depth of coverage, making it best suited to detect medium-size CNVs from low pass sequencing. This is consistent with the idea that moderate deviation of distances from expectation in multiple mate pairs in the same locus might provide statistically significant evidence for inferring small indels [13].

Here we describe a computational method for inferring medium-size deletions and other CNVs from low-pass WGS data, with a model to incorporate both depth of coverage and mate pair information.

## Results

### A Hidden Markov Model

**Figure 1:**
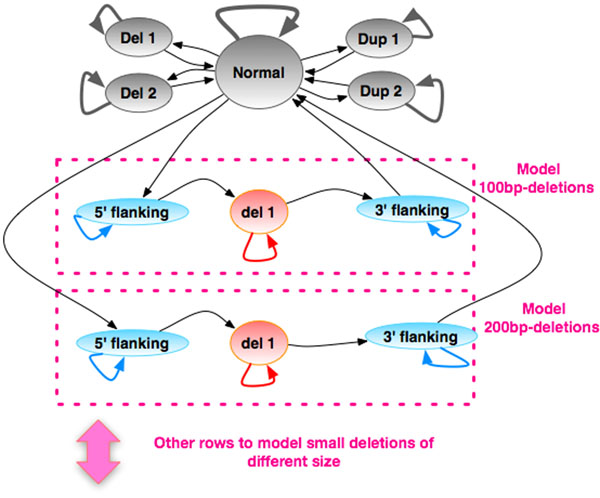
The Hidden Markov Model. The states: (1) Normal: normal diploid state, copy number 2, mate pair distance follows the empirical distribution observed from genome-wide data. (2) Del 1: 1-copy deletion state, mate pair distance follows the empirical distribution observed from genome-wide data. (3) Del 2: 0-copy deletion state, mate pair distance follows the empirical distribution observed from genome-wide data. (4) Dup 1: 3-copy duplication state, mate pair distance follows the empirical distribution observed from genome-wide data. (5) Dup 2: 4-copy duplication state. This state is also capable of capturing duplications with more than 4 copies. (6) Grid states: marked with purple dashed frames. Each row of grid states targets deletions of a particular size. The 5’ flanking and 3’ flanking states (collectively, *F* states) model the flanking regions of such deletions where the mate pair distances follow the empirical distribution with a shifted mean value (genome-wide average + deletion size).

The core algorithm is a Hidden Markov Model (HMM) [14], in which both depth of coverage and mate pair distances are used to calculate the emission probability. Depth of coverage correlates directly with copy number, following a theoretical Poisson distribution with genome-wide average as *λ*[15]. However, there are systematic biases in sequencing, which leads to overdispersion. Empirically, the overdispersion can be primarily explained by the GC bias [1], and the distribution can be modeled by an overdispersed Poisson or negative binomial [16,17]. Abnormality in mate pair distance, order, or orientation suggests breakpoint(s) from CNVs or other structural variations, and the distance reflects the size of a deletion or tandem duplication. The primary challenge of modeling mate pairs through a HMM is that a 1^st^ order Markov chain does not store the information of inferred CNV size once the path goes beyond one end of a pair of break points, and higher order Markov chain is computational prohibitive for processing genome-wide data. To address this issue, we augment a regular 1^st^ order HMM with a grid of specialized deletion states that explicitly model medium-size deletions and flanking regions (*F* states) (Figure 1). Specifically, in the generative model going through a chromosome by each position, a hidden state represents the copy number, emitting both number of reads starting from this position and the out-distance of the mate-pair. And the emission probability of a state in the HMM is calculated jointly from the mate-pair distance and depth of coverage (Equation 1).(1)

In Equation 1, *n* is the number of reads start at a genomic position, *d* is the out-distance between a pair of reads, *C* is the copy number of the state, *δ* is the shifted value of mean out-distance of the state, *λ* is the genome-wide average number of reads starting at any position, *G* is the depth of coverage adjustment value based on local GC content, and *D* is the genome-wide mean out-distance. *Pr*(*d* | *D*, *δ*) is the probability of *d* assuming the insert size follows the empirical distribution observed from data with mean shifted by *δ* (i.e., mean is *D*+*δ*). We assume *δ* is 0 for for all states except for *F* states, where *δ* is the approximate size of the deletions flanked by these states. The model targets deletions of different sizes by setting different *δ* values for flanking states on different rows.

The transition probabilities from normal to other states are set heuristically to reflect rough estimation of the number of medium- to large- size deletions and duplications in the genome. The transition probability of a non-normal state to itself is set to reflect the approximate duration of that state along the genome in a generative model, which for *F* states is the targeted size of the flanked deletion. Other transition probabilities are calculated based on the constraint that the total probability of all in- or out- transitions is 1. We use Viterbi algorithm to infer the most likely copy-number status path along a chromosome given the sequence data.

### Results from simulated data

**Figure 2:**
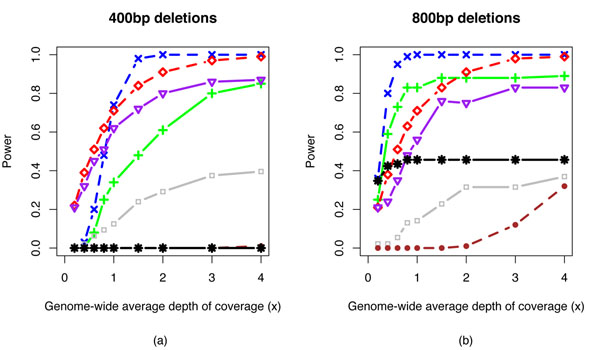
Power of detecting medium-size deletions at low to moderate depth of coverage. The blue and red dashed lines are theoretical values using 1.5kbp and 200bp insert libraries, respectively. The green and purple solid lines are results from Zinfandel on the simulated data with 1.5kbp and 200bp insert libraries, respectively. The standard deviation of 1.5kbp- and 200bp- inserts was set at 200 and 20, respectively, based on empirical data from Life SOLiD^TM^ and Illumina Solexa^TM^ platforms [17]. The black and gray lines are results from BreakDancer on the simulated data, and the brown dashed lines are theoretical values for methods solely based on depth of coverage.

We constructed a human diploid genome by duplicating the human reference genome, and then altering it with deletions and tandem duplications placed randomly. Aiming to demonstrate the power and resolution of our method, we focused on medium size CNVs (400 and 800bp) and only simulated heterozygous CNVs. Homozygous CNVs of comparable sizes are obviously easier to detect. We then simulated 0.2× ~ 4× haploid coverage shotgun reads [3] with fixed size at 35bp. We set two types of insert libraries, one with mean insert size 200bp (standard deviation of 20) and the other with mean insert size 1.5kbp (standard deviation of 200). We mapped [3] the simulated reads to the human reference genome. We applied our method to infer CNVs from the simulated data. A CNV is regarded as correctly identified if the inferred CNV and the simulated one have 50% mutual overlap. The power of detecting CNVs from simulated data is consistent with the theoretical values, and exceeds 0.8 at ≥ 3× depth of coverage for medium-size (400-800bp) CNVs (Figure 2). We did not observe false discoveries outside of known gaps in the reference genomes including centromeres. Figure 2 also shows the results of BreakDancer [11] on the simulated data. BreakDancer was less powered to detect deletions of size comparable to the shotgun insert library size from low-pass sequencing. Particularly, it was not able to detect any 400bp heterozygous deletions from 1.5kbp insert library.

## Discussion and conclusions

We described a Hidden Markov Model-based method that integrates the depth of coverage with mate-pair information from whole genome sequencing data to infer CNVs. Using simulation, we demonstrated that this method achieved near-theoretical power for detecting medium-size deletions from low-pass WGS data.

We applied BreakDancer [11] to the same set of simulated data and demonstrated that our method was better powered to detect medium-size deletions from low-pass sequencing. This is expected because BreakDancer infers CNVs based on discordant mate pairs that have larger outer-distance deviations than a fixed threshold. That makes it suboptimal in taking advantage of multiple discordant mate pairs that deviate from mean distance values consistently but less significantly than the threshold.

Medvedev et al 2010 [19] presented a donor graph- based method (CNVer) to infer CNVs using both mate-pair and depth-of-coverage information. The advantage of CNVer is that by using mate pair discordant and depth-of-coverage information jointly, it has better accuracy and sensitivity of detecting CNVs flanked by segmental duplications, which are the places where traditional mate-pair based methods have difficulties because of non-unique mapping of reads. Similarly to BreakDancer, CNVer requires an explicit out-distance cutoff (by default three times the standard deviation of insert library size) to establish a list of discordant mate pairs. Therefore, CNVer would have poor power to detect deletions with size close to the threshold values. Our method takes advantage of the fact that multiple mate-pairs with small but consistent deviations of outer-distances could still provide statistically significant evidence, and models it explicitly through “flanking” regions in the HMM. Additionally, CNVer requires combination of support from depth-of-coverage and mate pairs. This is effective for reducing false positives, but makes it difficult to detect small- or medium-size CNVs that are intrinsically underpowered in depth-of-coverage based methods given low to medium genome-wide average depth.

It is feasible to process low-pass sequencing data from cancer tissues for inferring amplification loci, which will be called 4-copy duplications in our method, and then estimate the amplification level by other methods based on the number of reads mapped to the regions.

One limitation of our method is that hemizygous deletions are not modeled. A future direction is to detect CNVs from exome data. Comparing to whole genome sequencing, exome sequencing requires an extra capture step, which makes the depth of coverage distribution much more overdispersed than Poisson, and therefore presents computational challenges in depth-based CNV detection. A reasonable approach would be to call CNVs jointly from multiple samples.

## Methods

### Theoretical power calculation

#### Power of detecting CNVs based on depth-coverage only

The depth-coverage from whole-genome shotgun sequencing follows Poisson distribution [15]. Due to various experimental issues, such as the unevenness of accessibility of sequencing from genomic structure and the DNA melting temperature dependence on local GC content [1], the real distribution is overdispersed, and can be modeled by a negative binomial or overdispersed Poisson [17].

**Figure 3:**
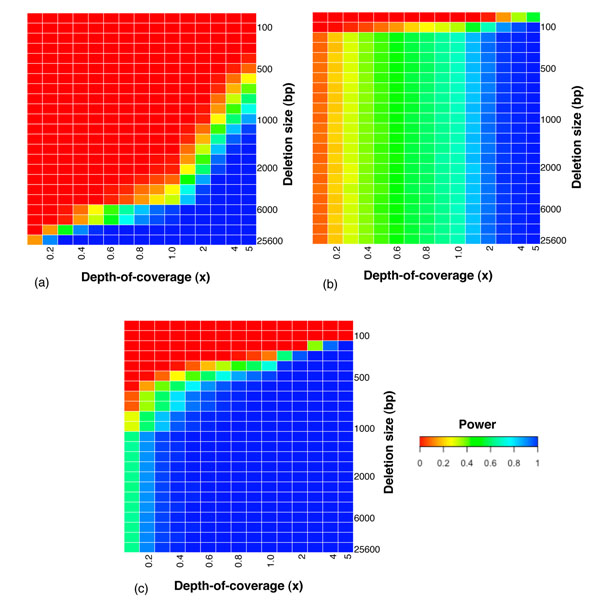
Theoretical power of detecting CNVs. Heat-map shows the dependence of power on deletion size (y-axis) and depth-coverage (x-axis). Read size S is fixed at 35bp. Type I error cutoff is 10^-5^. (a) Power of detecting CNVs based on depth-coverage only. The deletion size R is in the range of 50bp to 256Kbp. The depth-coverage *λ* is in the range of 0.1 to 5×. (b) Power of detecting CNVs based on mate-pair distance from insert library with mean 200bp and standard deviation 20. (c) Power of detecting CNVs based on mate-pair distance from insert library with mean 1500bp and standard deviation 200.

To illustrate the power and resolution of CNV detection from shotgun reads, we consider a simple Poisson model. Assuming the chromosome-wide average depth-coverage is *λ*, the read size is fixed at *S*, then in a region of size *δ* (*δ* >*S*), the number of shotgun reads sampled from a copy-neutral region follows Poisson distribution with mean *δ*·*λ*/*S*. Likewise, the number of reads from a 1-copy gain region follows a Poisson distribution with mean 1.5·*δ*·*λ*/*S*, and the number of reads from a 1-copy loss region follows a Poisson distribution with mean 0.5·*δ*·*λ*/*S*. Let copy-neutral be the null model, we can then calculate the power of detecting 1-copy gain or 1-copy loss with a pre-set type I error cutoff. Figure 3(a) shows the dependence of power on region size *δ* and average depth-coverage *λ*.

#### Power of detecting CNVs based on mate-pair information

The outer-distance of a mapped pair of reads reflects the insert size. If the insert contains a deletion, the mapped distance will be larger than the expected by the deletion size. To calculate the power of detecting a deletion of certain size based on mate pair distance, we define a null hypothesis and an alternative hypothesis. The null hypothesis is that the mapped distance of a pair follows the empirical distribution of all the mapped pairs from the same run, which is a valid approximation because the majority of the inserts are from genomic regions where the target genome does not contain deletions comparing the reference genome. Denote the mean of the empirical distribution as *D*, which is actually a point estimation of the insert library size. The alternative hypothesis is that the insert contains a deletion of size *δ*, therefore the mapped distance follows a distribution with same shape as the empirical distribution but with a different mean of *D*+*δ* . The power of detecting a deletion of *δ* based on a single pair of reads is: 1- *CDF*(*Q* | *D*+*δ* ), where *Q* is the quantile value of the null distribution (with mean value *D*) given *p* = type I error, *CDF*() is the cumulative density function of the alternative distribution (with mean value *D*+*δ*) at *Q*.

An optimal algorithm of detecting deletions do not require a single pair to have obvious outlier mapping distance, but can integrate the significance of the deviation from expectation from multiple pairs. To estimate the power of detecting a deletion based on multiple pairs, we approximate the null distribution of mapping distance using a Gaussian distribution with mean *D* and standard deviation *σ* (empirically the approximation is more accurate near *D*). Assume a deletion of size *δ* is contained in *m* inserts (thus *m* pairs of reads), the standard error of the mapped distances is . The power can be calculated based on Z-score: . Given genome-wide average depth-of-coverage *λ*, read size *S*, then the expected value of *m* is , therefore, . Figure 3(b) and (c) show the dependence of power on deletion size *δ* and average depth-coverage *λ* with two examples of insert libraries (*D* and *σ* values are based on empirical data from [17]).

### The Hidden Markov Model and implementation

The possible states in our HMM are: “normal”, which models copy neutral sites, “Del1”, which models heterozygous deletions, “Del2”, which models homozygous deletions, “Dup1”, which models three-copy duplications, and “Dup2”, which models four-copy duplications including homozygous duplications and heterozygous duplications composed of one normal copy and one three-copy duplications, and “5’ flanking” and “3’ flanking”, which model the 5’ and 3’ breaking points of deletions.

We use heuristics to set the transition probabilities. Specifically, the transition probability from a any state to itself is the inverse of the expected duration of the state. For the deletions and duplications that directly connected with Normal state, the expected duration is average size of the CNVs that are well powered for detection based on depth-of-coverage. The expected duration of deletion states in the “grid” is the targeted deletion size of the row where the state is located (200bp, 400bp, 600bp, 800bp, …, and 1600bp). The expected duration of the 5’ and 3’ flanking regions is the mean size of the insert library. The duration of the Normal state is estimated by dividing the genome size with the expected total number of CNVs. The transition from a state to other states is constrained by the model structure, and the probability is equally distributed among the destination states.

We implemented the algorithm in Java. Running the program on a commodity Linux server to process a human genome sequenced at 4× requires about thirty hours CPU time. In practice, it is convenient to split the genome into overlapping fragments and carry out CNV detection on different fragments in parallel to speed up the process.

### Parameters

The input data of Zinfandel is the reads-alignment output in “mapview” fomat from maq [3]. Support for SAM/BAM format [18] is planned for future version. The maq mapping parameters are all default except –a (outer-distance outoff), which was set to 6000.

BreakDancer parameters were set to default of breakdancer-max version 1.0. Deletions called by BreakDancer were filtered based on score using a cutoff of 40 as recommended by the program.

### Definition of outer-distance

Assume the mate pair insert library was prepared in forward-reverse (FR) orientation [3]. Denote the mapping position of 3’ end of the read with negative strand as E, and the 5’ end of the read with positive strand as S, then the out-distance is defined as *D*≡*E*-*S*.

## Competing interests

The authors declare that they have no competing interests.
